# Supplementary material for: An osmium-peroxo complex for photoactive therapy of hypoxic tumors
Source: Nat Commun. 2022 Apr 26;13:2245. doi: 10.1038/s41467-022-29969-z (PMC9042834; doi:10.1038/s41467-022-29969-z)
Supplement: Supplementary file 1 — Supplementary Information [file 41467_2022_29969_MOESM1_ESM.pdf]

Supplementary information

An osmium-peroxo complex for photoactive therapy of hypoxic tumors

Nong Lu,[a], ‡ Zhihong Deng,[b], ‡ Jing Gao,[b, c] Chao Liang,[a] Haiping Xia,* [b] and Pingyu Zhang*[a]

[a] N. Lu, Dr. C. Liang, Prof. Dr. P. Zhang, College of Chemistry and Environmental Engineering, Shenzhen University, Shenzhen, 518060, China. E-mail: p.zhang6@szu.edu.cn (P. Zhang)

[b] Dr. Z. Deng, Dr. J. Gao, Prof. Dr. H. Xia, Shenzhen Grubbs Institute, Department of Chemistry, Southern University of Science and Technology, Shenzhen 518055 China. E-mail: hpxia@xmu.edu.cn (H. Xia)

[c] Dr. J. Gao, Center for Reproductive Medicine, the Third Affiliated Hospital of Sun Yat-sen University, Sun Yat-sen University, Guangzhou, 510630, China.

‡These authors contributed equally.

Contents

Supplementary Methods

Supplementary Figures

Supplementary Figure 1. The ESI-MS spectrum of **Os2**.

Supplementary Figure 2. The ^{1}H NMR spectrum of **Os2**.

Supplementary Figure 3. The ^{13}C NMR spectrum of **Os2**.

Supplementary Figure 4. The stability of **Os2** in the PBS in the dark.

Supplementary Figure 5. The HR-MS spectrum of **Os2** after illumination.

Supplementary Figure 6. The ESI-MS spectrum of **Os1**.

Supplementary Figure 7. The ^{31}P NMR spectrum of **Os1**.

Supplementary Figure 8. The ^{1}H NMR spectrum of **Os1**.

Supplementary Figure 9. The ^{13}C NMR spectrum of **Os1**.

Supplementary Figure 10. X-ray structure of **Os1**.

Supplementary Figure 11. The transform rate of **Os2** under light irradiation.

Supplementary Figure 12. The UV-Vis absorption spectra of MB for monitoring of OH•.

Supplementary Figure 13. $^1O_2$ generation of **Os1-Os2** or $[Ru(bpy)_3]^{2+}$ measured by ABDA probe.

Supplementary Figure 14. $O_2^{\bullet-}$ generation of **Os2** in hypoxia.

Supplementary Figure 15. ROS measurement in hypoxia.

Supplementary Figure 16. The stability of **Os2** in the DMEM media in the dark.

Supplementary Figure 17. The UV-Vis absorption spectra of **Os2** in the different pH solution.

Supplementary Figure 18. The stability of **Os2** in the presence of different reducing agents or cell oxidants.

Supplementary Figure 19. The UV-Vis absorption spectra of ABDA in the presence of Ce6 and **Os2** in the dark or under 633 nm light irradiation.

Supplementary Figure 20. The UV-Vis absorption spectra of **Os2** in PBS solution containing Ce6 in the dark or under 633 nm light irradiation.

Supplementary Figure 21. The cellular $^1O_2$ generation.

Supplementary Figure 22. Cellular uptake of **Os2** or cisplatin in HeLa cells measured by ICP-MS.

Supplementary Figure 23. Fluorescence microscopy images of the HeLa cells treated with **Os2** and co-stained with calcein AM and propidium iodide after different treatments.

Supplementary Figure 24. UV-Vis absorption spectra for the photocatalytic oxidation of NADH by **Os2**.

Supplementary Figure 25. UV-Vis absorption spectra for the photocatalytic oxidation of NADH by **Os1**.

Supplementary Figure 26. H&E staining images of the major organs of the treated mice.

Supplementary Figure 27. The images of zebrafish larvae after treatment with **Os2**.

Supplementary Tables

Supplementary Table 1. Selected bond lengths and bond angles for **Os1**.

Supplementary Table 2. Crystal data and structure refinement for **Os1**.

Supplementary Table 3. The singlet oxygen quantum yields (Φ) of **Os1** and **Os2**.

Supplementary References

Supplementary Methods

Materials. 9,10-anthracenediyl-bis-(methylene) dimalonic acid (ABDA), 2,2,6,6-tetramethylpiperidine (TEMP), 5,5-dimethyl-1-pyrroline-N-oxide (DMPO), methylene blue (MB), 1,4-dihydro-nicotinamide adenine dinucleotide (NADH), 5-aminolevulinic acid (5-ALA) and cisplatin were purchased from Sigma-Aldrich. Hydroxyphenyl fluorescein (HPF) and Ferrostatin-1 were purchased from Aladdin. C11-BODIPY was purchased from Cayman Chemical. Singlet Oxygen Sensor Green (SOSG), Dihydrorhodamine 123 (DHR123), 3-(4,5-dimethylthiazol-2-yl)-2,5-diphenyltetrazolium bromide (MTT) and Calcein AM /PI kit were obtained from Life Technologies. Dihydroethidium (DHE) and GSH and GSSG Assay kit were obtained from Beyotime Biotechnology. HeLa cell line were purchased from American type culture collection (ATCC). Dulbecco's modified eagle medium (DMEM), fetal bovine serum (FBS), glutamine and penicillin/streptomycin were purchased from Sigma-Aldrich.

Instruments. NMR spectra were recorded on a BrukerAV III-400/600 MHz spectrometer. HRMS measurements were conducted using Thermo Scientific Q Exactive instrument. Single-crystal X-ray diffraction data were collected using a Bruker APEX-II CCD diffractometer with CuKα radiation (λ = 1.54178 Å). Elemental analyses were performed on a Vario EL III elemental analyzer. UV-visible absorption spectra were recorded on a Hitachi UV-2500 spectrophotometer. The emission spectra were recorded on an Edinburgh FS5 Spectrofluorometer. HPLC spectra was recorded on Agilent 1260 high performance liquid chromatograph. ESR spectra were recorded using

a Bruker Model A300 ESR spectrometer equipped with a Bruker ER 4122 SHQ resonator. Confocal images were recorded on a Zeiss LSM 880 confocal microscopy. The cell viability assays were recorded using a Promega microplate reader. 465 nm LED lamp was used to generate light during the treatment. All ploted and calculated statistical analyses were performed on Excel 2019 and Origin 8.5.

Synthesis of Os2. The **Os2** was synthesized according to the reported method,[1] and was fully characterized by HRMS, ^{1}H NMR, ^{13}C NMR (Supplementary Figures 1-3) and elemental analysis. Elemental Analysis Calculated (%) for $C_{88}H_{74}BO_5OsP_3$: C 70.21, H 4.95; Found: C 70.53, H 4.94.

Synthesis of Os1. $NaNO_2$ (69 mg, 1 mmol) was added to a DCM solution of osmapentalyne[2] (119 mg, 0.1 mmol), and the solution was stirred at room temperature in air for 2 hours. After that, the solvent was removed under reduced pressure, and the resulting residue was purified by column chromatography on neutral alumina (dichloromethane/methanol, 30/1) to give **Os1** as a red solid (82 mg, 68% yield). ^{1}H-NMR (600.1 MHz, CH_2Cl_2): δ (ppm) = 12.90 (s, 1H, H7), 8.17 (d, $J_{\text{P-H}}$ = 4.3 Hz, 1H, H3), 8.07 (s, 1H, H5), 3.45 (s, 3H, COOC*H₃*), 7.73-7.01 (m, 45H, Ph). ^{31}P-NMR (242.9 MHz, CD_2Cl_2): δ (ppm) = 9.44 (s, C*P*Ph_3), 2.69 (s, Os*P*Ph_3). ^{13}C-NMR (150.9 MHz, CD_2Cl_2): δ (ppm) = 231.82 (m, C7), 223.44 (m, C1), 178.78 (dt, $J_{\text{P-C}}$ = 23.5 Hz, $J_{\text{P-C}}$ = 3.7 Hz, C4), 170.61 (d, $J_{\text{P-C}}$ = 14.1 Hz, C3), 161.28 (s, *C*OOCH_3), 157.84 (s, C5), 145.46 (s, C6), 117.53 (d, $J_{\text{P-C}}$ = 92.0 Hz, C2), 50.37 (s, COO*C*H3), 134.67-127.31 ppm (m, Ph). HRMS (ESI): *m/z* cacld for $[C_{63}H_{51}ClO_3OsP_3]^+$, 1175.2348; found, 1175.2355. Elemental Analysis Calculated (%) for $C_{63}H_{51}Cl_2O_3OsP_3$: C 62.53, H 4.25; Found: C 62.94, H 4.34. The crystal structure of **Os1** was in the Cambridge Crystallographic Data Centre (CCDC 1913382).

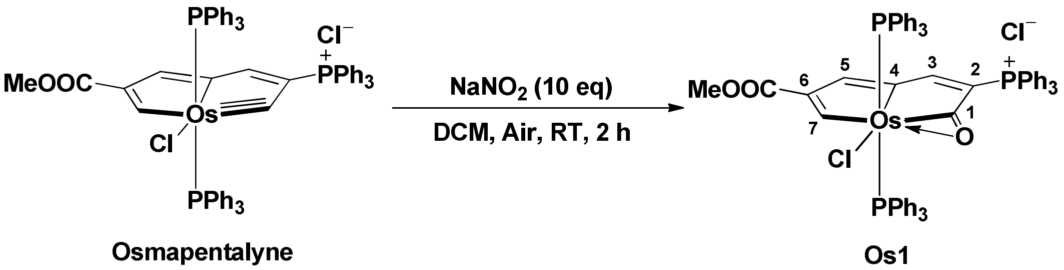

ESR measurements. ESR measurements were carried out on a Bruker Model A300 ESR spectrometer equipped with a Bruker ER 4122 SHQ resonator, using 1.0 mm quartz tubes. The TEMP and DMPO were used to detect $^1O_2$ and $O_2^{\bullet-}$, respectively. 20 μL TEMP (40 mM) or DMPO (90 mM) was mixed with 80 μL **Os2** (5 mM) or **Os1** (5 mM) and irradiated by light (465 nm, 13 mW/cm^2) for 1 h. As a comparison, the **Os2** or **Os1** mixed with TEMP or DMPO in the dark were detected as well.

Cell culture. HeLa cells were cultured in DMEM medium supplemented with 10% fetal bovine serum, 1% v/v glutamine and 5% v/v ciprofloxacin hydrochloride solution at 37 °C in a humid 5% CO_2-containing atmosphere. Mitsubishi™ AnaeroPack-Anaero (Japan) gas generator was used for cultivation of cells under hypoxic environment. Oxygen indicator was used to detect the oxygen levels in the chamber (1 % O_2).

Photo-toxicity and dark-toxicity in vitro. 5×10^3 cells/well HeLa cells were incubated with different concentrations of **Os2** or **Os1** for 8 h under hypoxia or normoxia. After that, the culture media was replaced by fresh culture media, which did not contain the complex. The 96-well plates of the dark groups were kept in the dark for 1 h. The 96-well plates of the light groups were irradiated by 465 nm light (13 mW/cm^2) for 1 h. After irradiation, upon further incubation for 40 h and then MTT (25 μL/well, 5 mg/mL) was used to stain the viable cells in the plates for 4 h. DMSO (150 μL/well) was added and the optical density was measured at 490 nm by a Promega microplate reader after shaking gently. The wells containing cells incubated without complex were set as control. The cell viability rate (VR) was calculated according to the equation: VR = (A-A_0)/(A_S-A_0)*100%, where A is the absorbance of the experimental group, A_S is the

absorbance of the control group and A_0 is the absorbance of the blank group (no cells).

Apoptosis assay. The live and dead assay was measured using calcein-AM and PI co-staining. HeLa cells (5×10^3 well^{-1}) were seeded in 96-well plates and incubated overnight. The cells were then incubated with/without **Os2** (20 μM) for 8 h. After that, the cells were treated or not by light irradiation (465 nm, 13 mW/cm^2, 1 h). After irradiation, upon further incubation for 40 h. The cells were stained with calcein-AM (4 μM, 0.5 h) for the visualization of live cells and with PI (6 μM, 0.5 h) for the visualization of dead/late apoptotic cells according to the manufacturer's suggested protocol.

Depletion of GSH in solution. The consumption of GSH was monitored by UV-Vis spectroscopy. **Os2** (20 μM) was mixed with GSH (200 μM) at room temperature. At different irradiation time points, 50 μL of this solution was added into 450 μL PBS, and then 2 μL 5, 5'-dithiobis-(2-nitrobenzoic acid) (DTNB) (10 mg/mL) was added. Then the absorbance spectrum of the supernatant was measured by UV-vis spectroscopy.

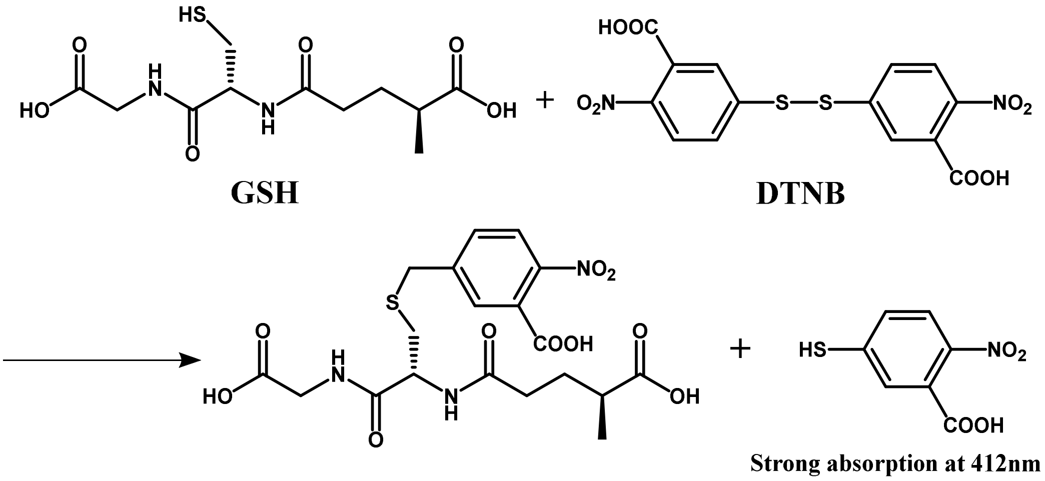

Cellular GSH detection. The **Os2**-induced GSH concentration variation was determined by using a GSH and GSSG Assay Kit (Beyotime). The HeLa cells (1×10^7) in a 13 cm culture dish were treated by **Os2** at a dose of 20 μM or 10 μM in the dark or under light irradiation (465 nm, 13 W/cm^2, 1 h). The incubation time was set at 8 h and drugs-free cells were used as the control. Then the cellular GSH concentrations of five

groups (20 μM (**Os2**-Dark and **Os2**-Light), 10 μM (**Os2**-Dark and **Os2**-Light) and Control) were measured according to the manufacturer's suggested protocol.

Intracellular lipid peroxides measurement. HeLa cells were incubated with 20 μM **Os2** for 8 h followed by incubation with 30 μM C11-BODIPY for 30 min (Fer-1(10 μM) was used as a control and incubated with **Os2** for 8 h). After that, cells were washed with PBS and then irradiated with 465 nm blue light for 1 h at a power density of 13 mW/cm^2. The green fluorescence was immediately observed using CLSM with the excitation wavelength of 488 nm, and emission collection wavelength from 520 nm to 620 nm.

GPX4 analysis. HeLa cells were seeded in 6-well plates at a density of 4 × 10^5 per well. After 24 h, **Os2** were added at a dose of 20 μM for 8 h incubation, the cells were irradiated (465 nm, 13 mW cm^{-2}) for 1 h. The cells without light irradiation were used as control and RSL3 (10 μg/ml) was a positive control. All cells were collected. The expression of GPX4 in HeLa cells upon formulation treatment was analyzed by western blotting according to the protocol method. The cell lysates containing identical protein (40 μg) were subjected to standard electrophoresis, followed by antibody incubation at 4 °C. The dilution ratio for the first antibody was 1:2000 (*β*-actin-specific antibody) and 1:2500 (GPX4-specific antibody). Regarding the secondary antibody, the dilution ratio was 1:5000 for both GPX4 and *β*-actin. The protein bands were developed via the ECLTM western blotting detection reagents.

Photocatalytic oxidation of NADH by UV-visible spectroscopy. **Os2** was evaluated for the catalytic oxidation of NADH to NAD^+ by UV-visible spectroscopy in the dark and under light irradiation (465 nm, 13 mW/cm^2) at 298 K. The **Os2** concentration remained fixed at 20 μM and the NADH concentration was 175 μM. The conversion of NADH to NAD^+ was followed by absorption at 339 nm (ε(NADH) = 6220 cm^{-1} M^{-1}) to allow evaluation of kinetic data. The catalytic turnover number (TON) was calculated using the following equations (1) and (2):

$$[NAD^+] = \frac{[Abs(339nm)_{initial} - Abs(339nm)_{final}]}{\text{Abs(339nm)}_{\text{initial}}} \times NADH \qquad (1)$$

$$\text{Turnover number } = [NAD^+] \div [Catalyst] \qquad (2)$$

Photocatalytic oxidation of NADH by ^{1}H NMR spectra. NADH (3.5 mM) was added to an NMR tube containing 0.25 mM of **Os2** in 66.6% CD_3OD/33.3% D_2O. ^{1}H NMR spectra of the resulting solutions were recorded at 298 K after 1 h light irradiation (465 nm, 13 mW/cm^2) or in the dark.

Cellular NADH sonocatalytic oxidation. The cells were treated under four different conditions (control, Light alone, **Os2**-Dark, **Os2**-Light), **Os2**: 20 µM. Then the cellular NADH concentrations were measured by using a NAD/NADH-Glo kit (Promega) according to the manufacturer's suggested protocol.

Photoactive therapy in vivo. Female BALB/c mice (6-8 weeks) were used as animal model in this work and were purchased from Liaoning Changsheng Biotechnology Co. Ltd. Mice were housed in individually ventilated cage (IVC) systems (ambient temperature: 23 ± 3 °C; relative humidity: 40-70%) and exposed to a 12-h light–dark cycle with free access to food and water. This work was conducted in according with Animal Care and Institutional Ethical Guidelines in China. And all animal experiments were carried out under the permission by the Ethic Committee of Shenzhen University (certificate number: SYXK 2014-0140). One million HeLa cancer cells in 25 µL PBS were subcutaneously injected to the right back of each mice. About 7 days after injection, the mice with ~100 mm^3 tumor volume were selected for further experiments.

To examine in vivo tumor growth inhibition by **Os2**, we randomly divided the HeLa tumor bearing Balb/c mice into 4 groups (n = 5 in per group). i) control dark group with only PBS injection; ii) Control light irradiation group (PBS injection, 465 nm, 13 mW/cm^2, 1 h); iii) **Os2**-Dark group (i.t. injection, 25 µL, 500 µM); iv) **Os2**-Light irradiation group (i.t. injection, 25 µL, 500 µM, 465 nm, 13 mW/cm^2, 1 h). Light irradiation was carried out on the zero day and record the size of the tumor every two

days. Tumor volume was calculated using the following formula: volume = ((tumor length) * (tumor width)^2)/2. At the end of experiment, the mice in different groups were sacrificed and their tumors were collected for photographing and weighing. After treating the tumors with different treatments, they were used for H&E staining on the next day.

Biosafety evaluation. For long-time toxicity evaluation, healthy Balb/c mice were intravenously injected with **Os2** (2.69 mg kg^{-1}) and sacrificed at various time points (the 1st and 7th day) after injection. Untreated healthy Balb/c mice were chosen as control. Any mouse of each group was sacrificed and the main organs (heart, liver, spleen, lung and kidney) were obtained for H&E staining histological examination.

The zebrafish (Tg(flk1:EGFP)s843[3]) was given by the Center of Experiment Animals at Sun Yat-Sen University. All zebrafish has been transfected by GFP. Embryos and zebrafish were housed in circular petri dish (Case of 150) at 28 ± 2 °C, and monitored at least once a day. 12-well plates were prepared with 3 concentrations of **Os2** (1 μM, 5 μM, 10 μM) in triplicate; DMSO used to solubilize complex did not exceed 1% v/v in final solutions and the control wells were set as well (control: 1% DMSO). The plates were seeded using at least 5 zebrafish per well, then incubated for 5 day. Then the individual zebrafish mortality and the fluorescence of GFP were assessed using an Olympus XI51 microscopy. GFP: λ_{ex} = 460 nm; λ_{em} = 510-550 nm.

Supplementary Figures

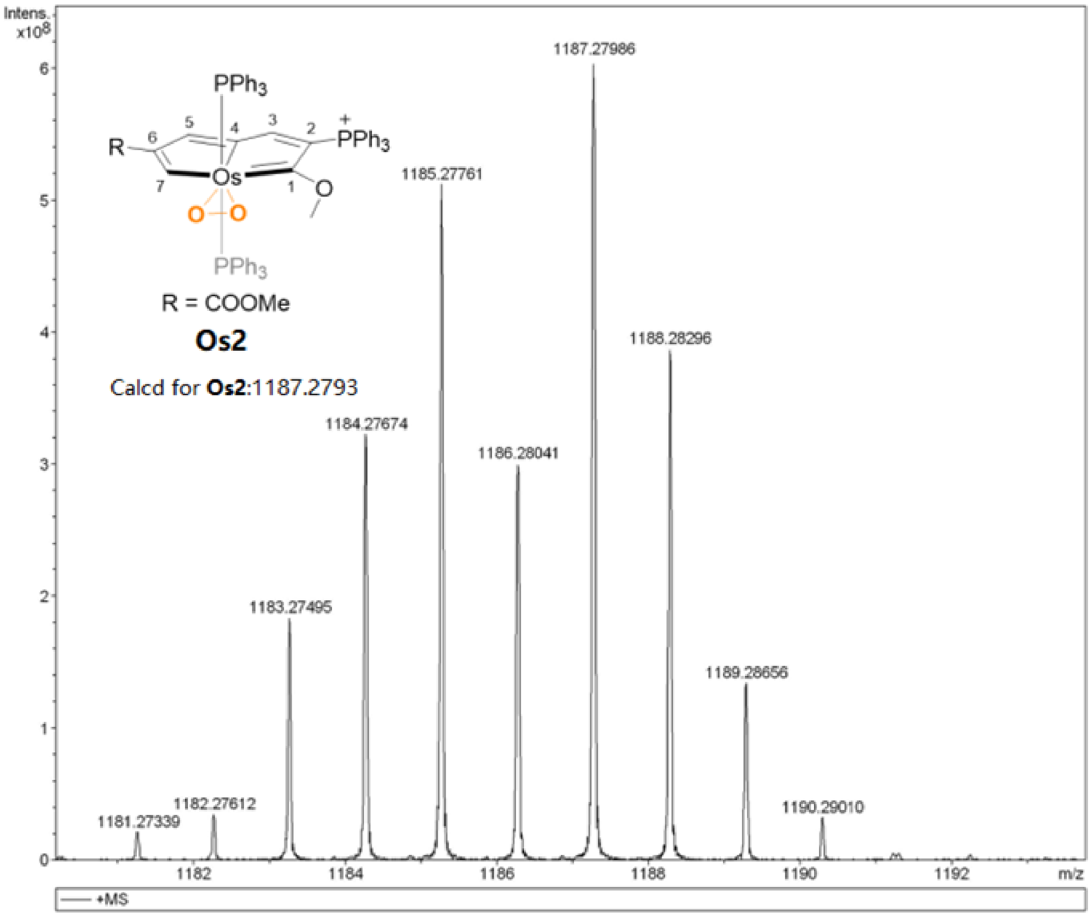

Supplementary Figure 1. Positive-ion ESI-HRMS spectrum for **Os2** measured in methanol.

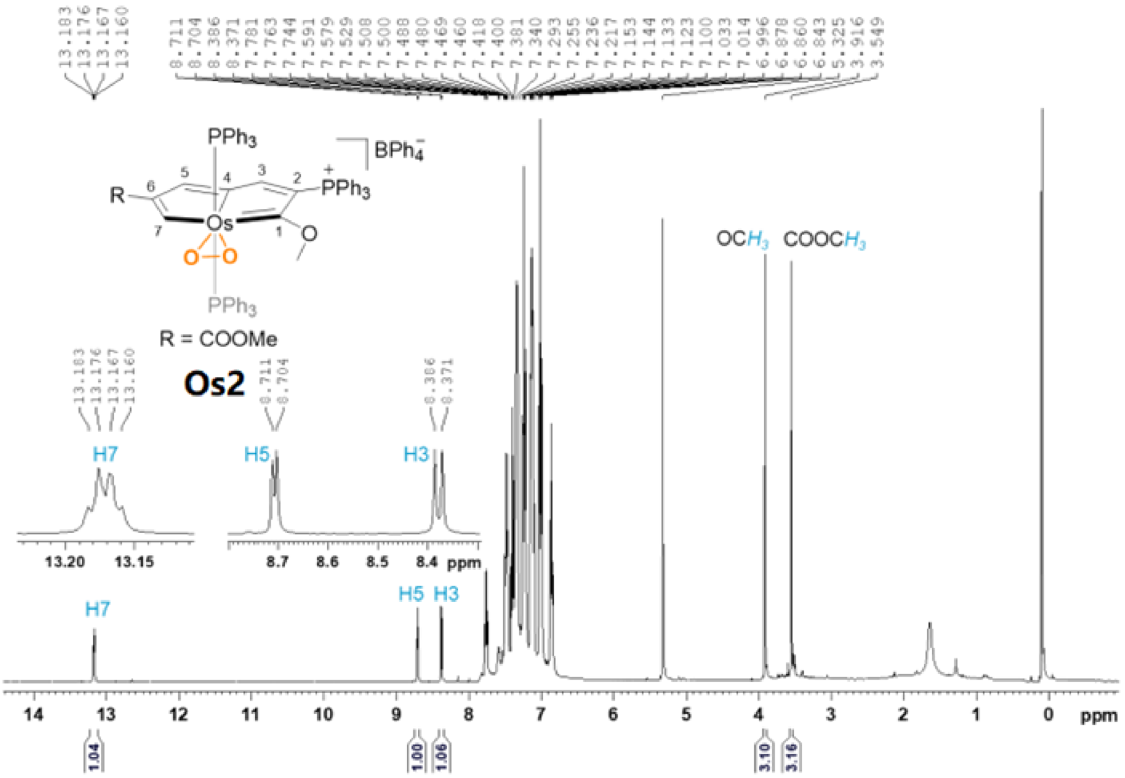

Supplementary Figure 2. The ^{1}H NMR (400.1 MHz, CD_2Cl_2) spectrum for **Os2**.

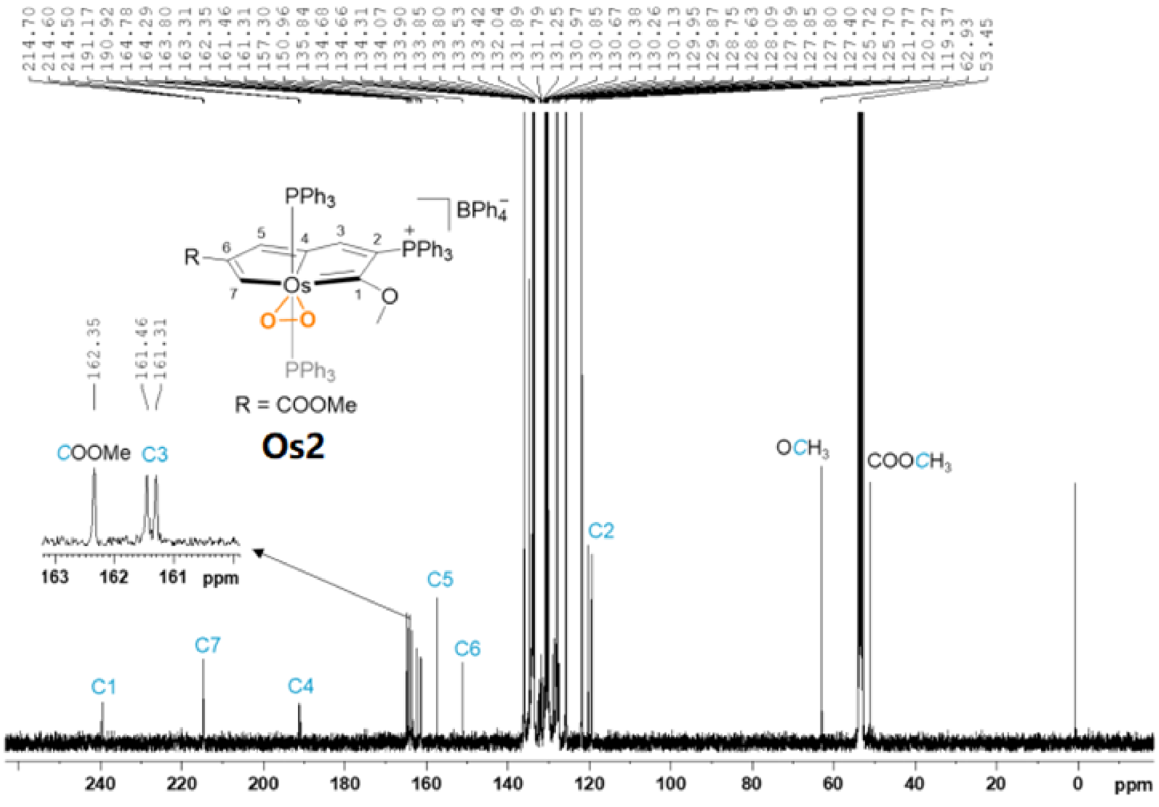

Supplementary Figure 3. The ^{13}C NMR (100.6 MHz, CD_2Cl_2) spectrum for **Os2**.

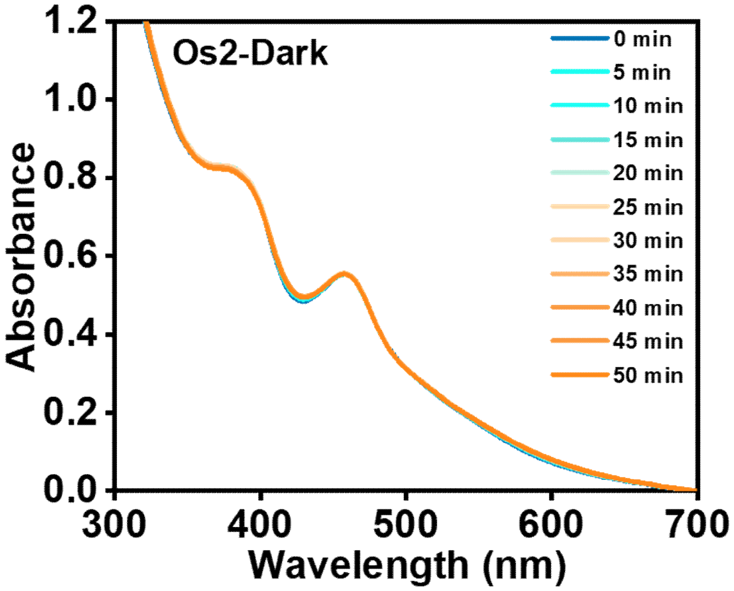

Supplementary Figure 4. The UV-Vis absorption spectra of **Os2** (100 μM) in PBS solution (pH 7.4) in the dark at 298 K.

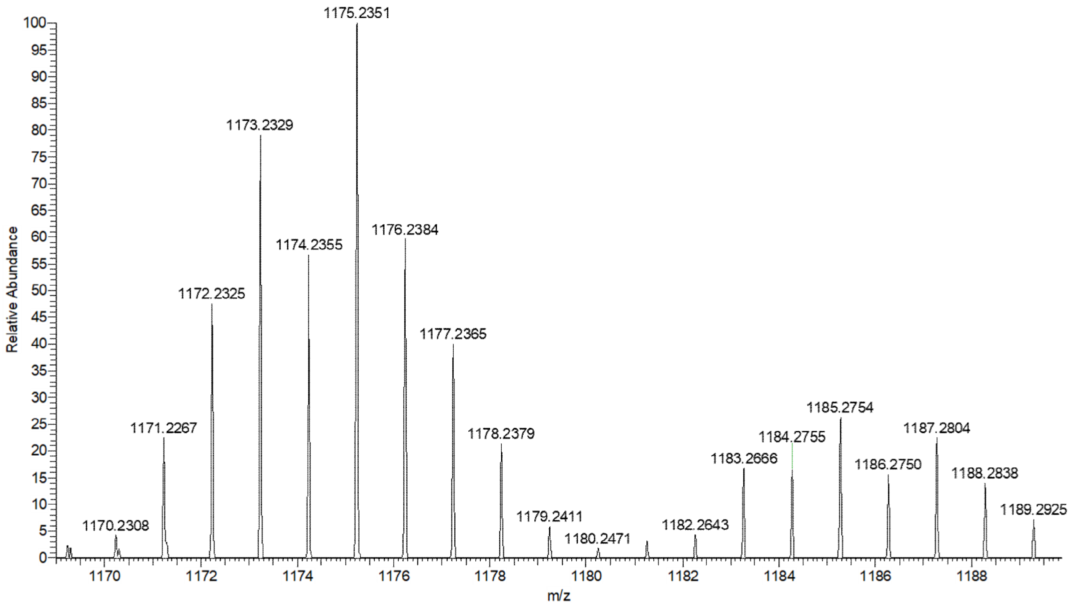

Supplementary Figure 5. The HR-MS spectrum of **Os2** after light illumination. Take 100 μM **Os2** in water containing 200 μM Cl^- after 1 h 465 nm light irradiation (13 mW/cm^2) to measure HR-MS spectrum.

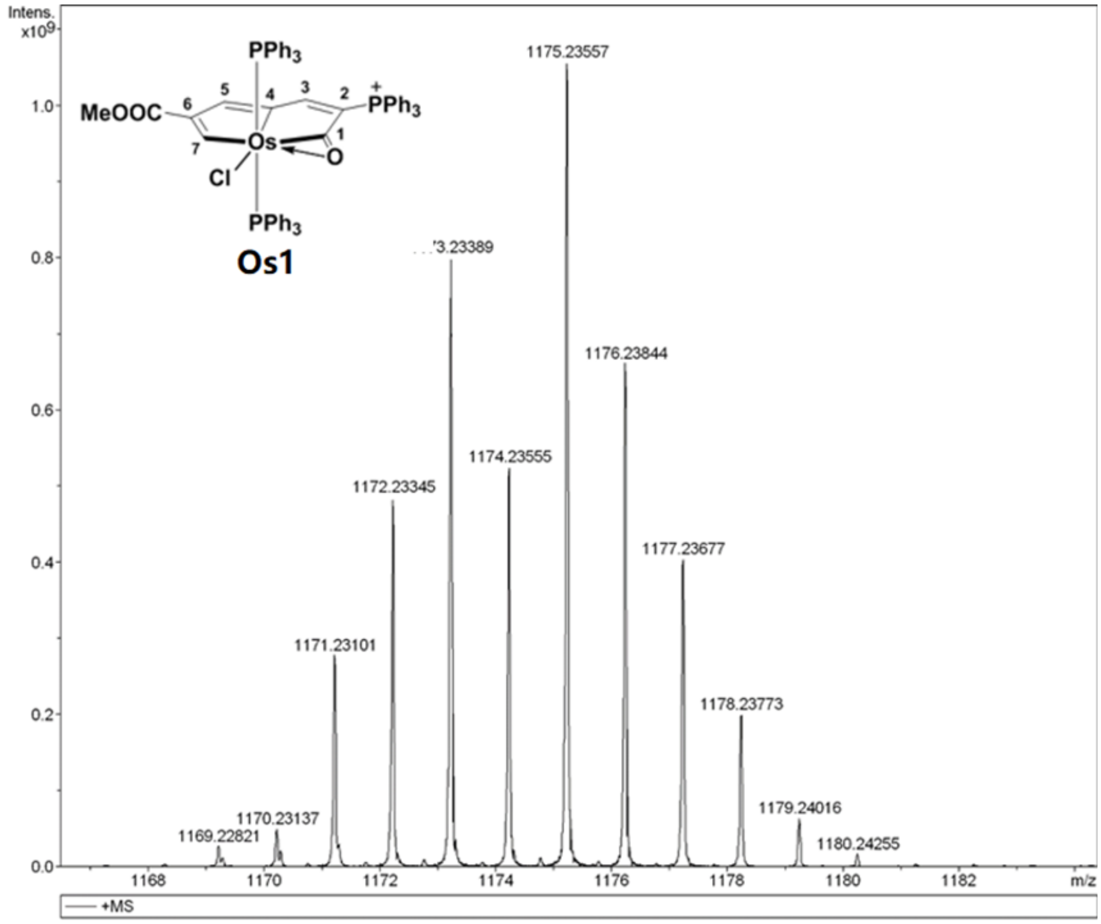

Supplementary Figure 6. Positive-ion ESI-MS spectrum for **Os1** measured in methanol.

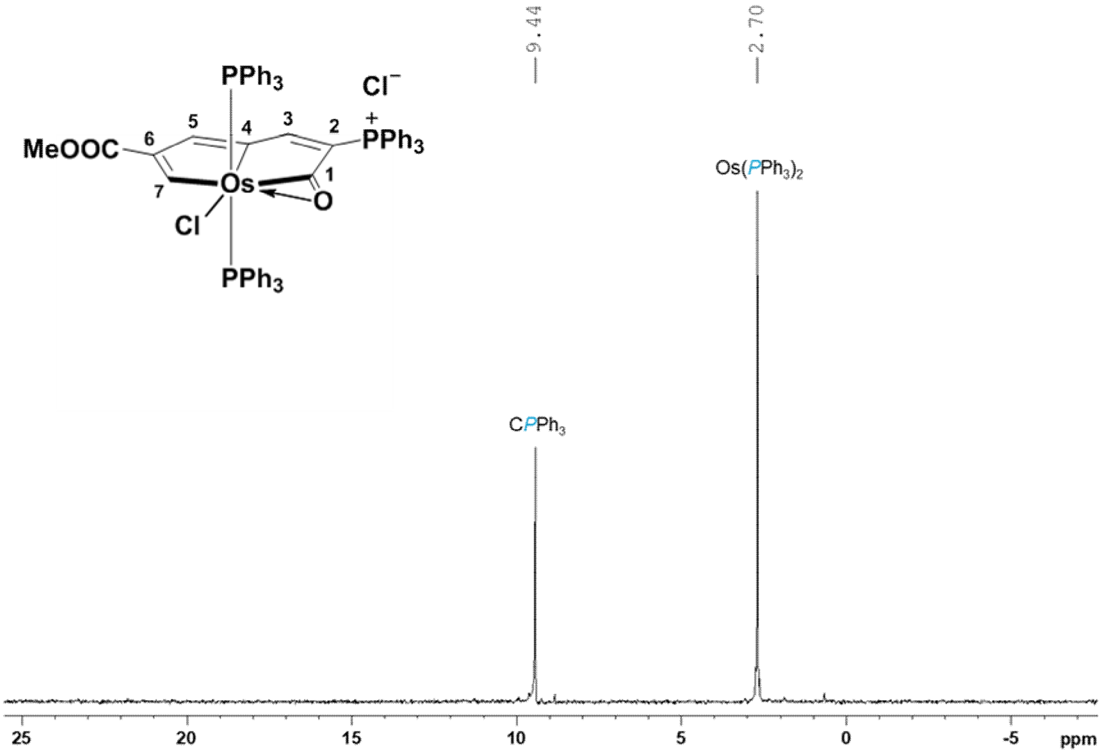

Supplementary Figure 7. The ^{31}P NMR (242.9 MHz, Dichloromethane-d_2) spectrum of **Os1**.

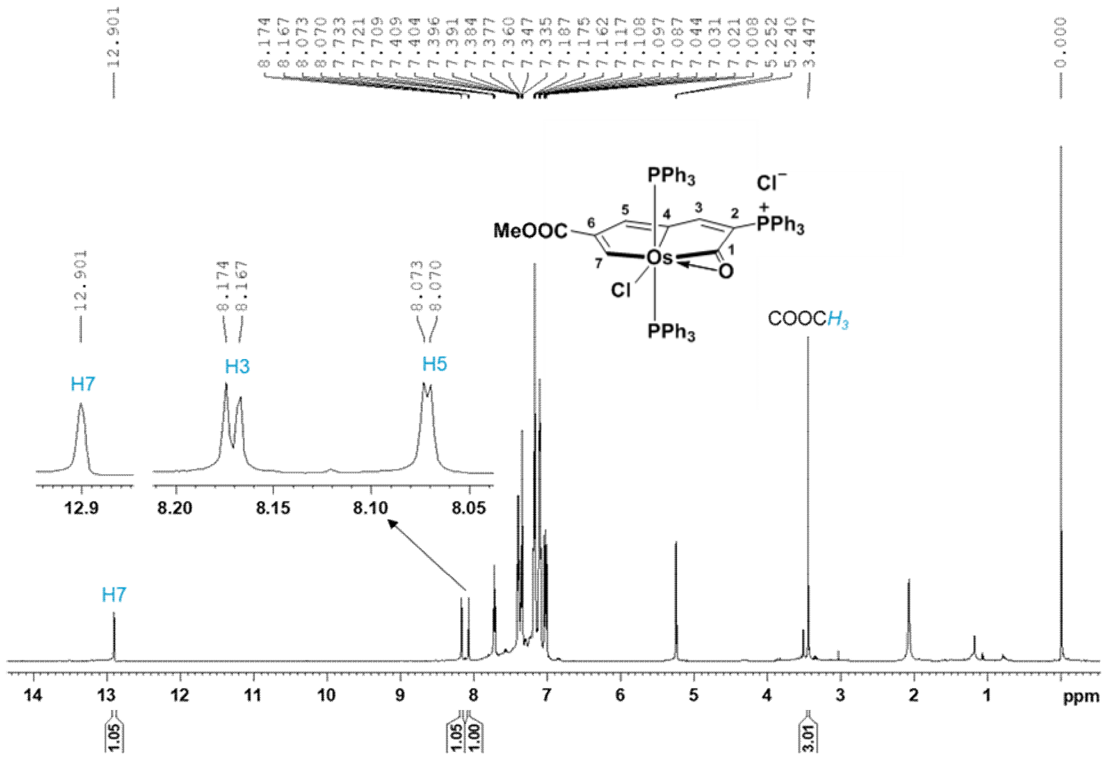

Supplementary Figure 8. The ^{1}H NMR (600.1 MHz, Dichloromethane-d_2) spectrum of **Os1**.

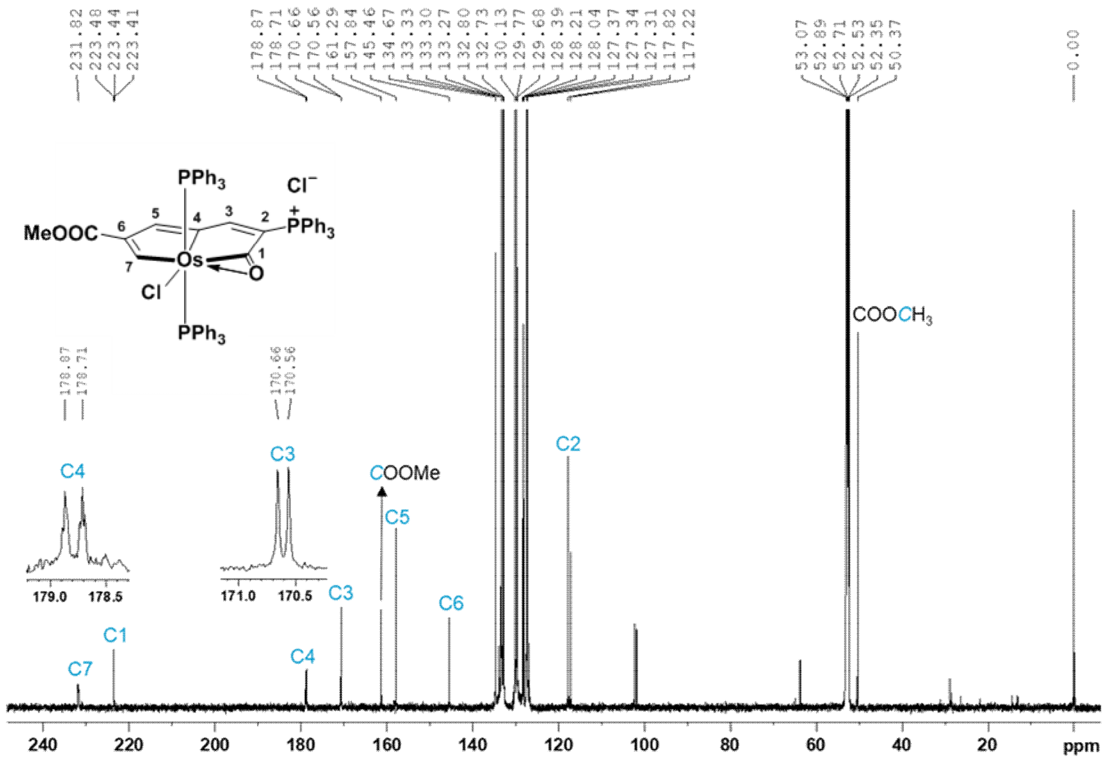

Supplementary Figure 9. The $^{13}C\{^{1}H\}$ NMR (150.9 MHz, Dichloromethane-d_2) spectrum of **Os1**.

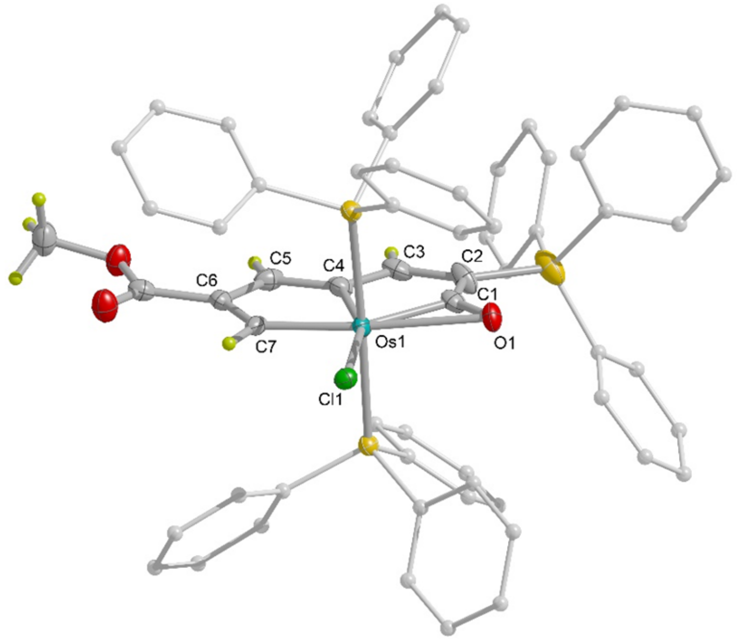

Supplementary Figure 10. X-ray structure of the cation of **Os1** with thermal ellipsoids drawn at 50% probability level. Hydrogen atoms in the triphenylphosphine moieties are omitted for clarity.

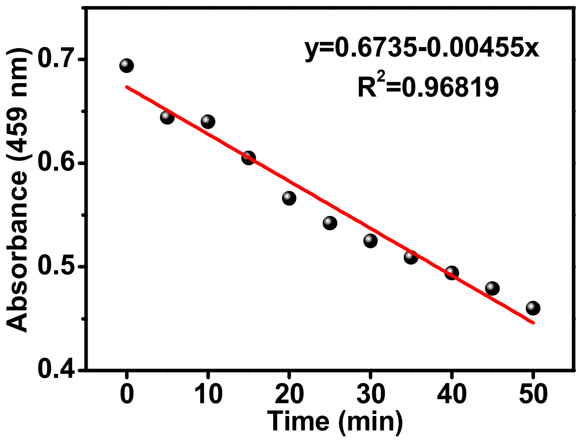

Supplementary Figure 11. The transform rate of the band at 459 nm for **Os2** under light irradiation in **Fig. 1b**.

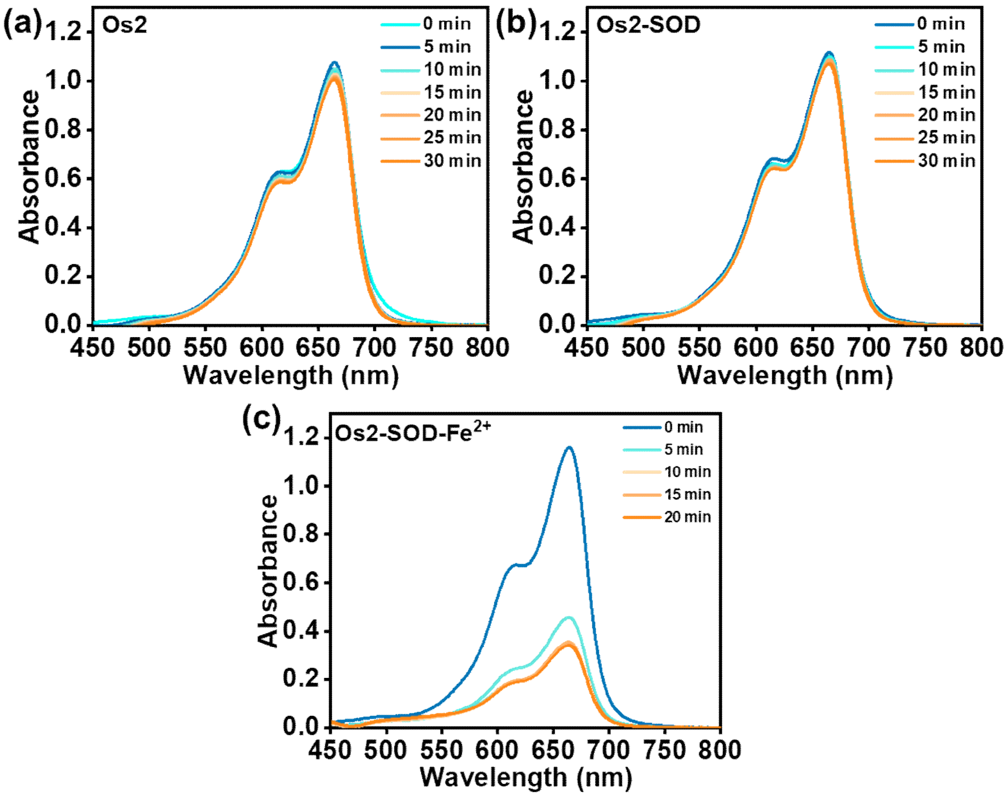

Supplementary Figure 12. The UV-Vis absorption spectra of MB (5 μg/mL) for monitoring of OH• generation in the (a) **Os2**, (b) **Os2**+SOD solution or (c) **Os2** in the Fe^{2+} (0.2 mM) solution containing SOD under light irradiation at 298 K. Light: 465 nm, 13 mW/cm^2; **Os2**: 15 μM.

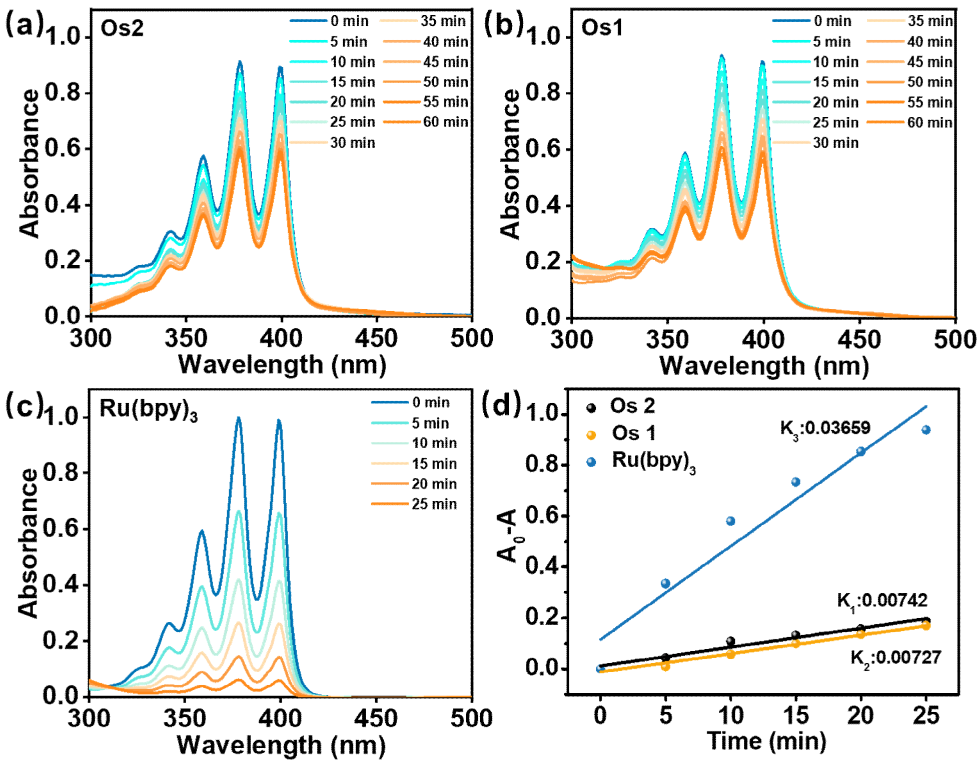

Supplementary Figure 13. The UV-Vis absorption spectra of ABDA (100 μM) in the presence of 20 μM (a) **Os2**, (b) **Os1** or (c) $[Ru(bpy)_3]^{2+}$ for monitoring of $^1O_2$ generation under light irradiation in normoxia at 298 K. (d) Rate constant for $^1O_2$ generation according to the absorbance of ABDA at 378 nm in (a), (b) and (c). Light: 465 nm, 13 mW/cm^2.

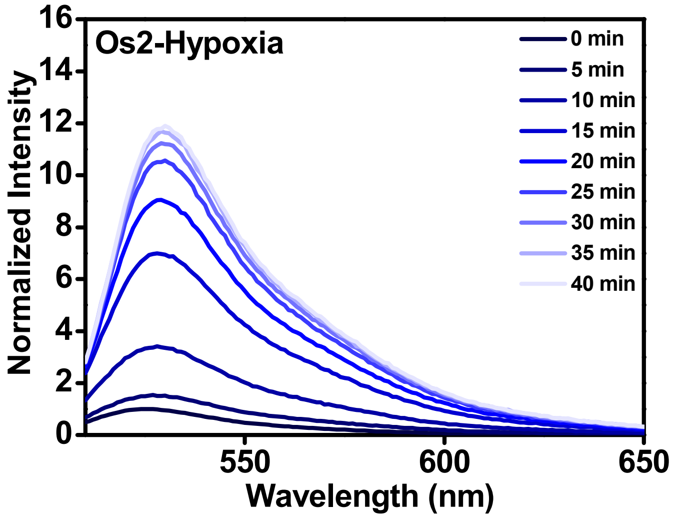

Supplementary Figure 14. The emission spectra for monitoring of $O_2^{\bullet -}$ generation by **Os2** (10 μM) using DHR 123 probe (10 μM, λ_{ex} = 488 nm) under hypoxia at 298 K.

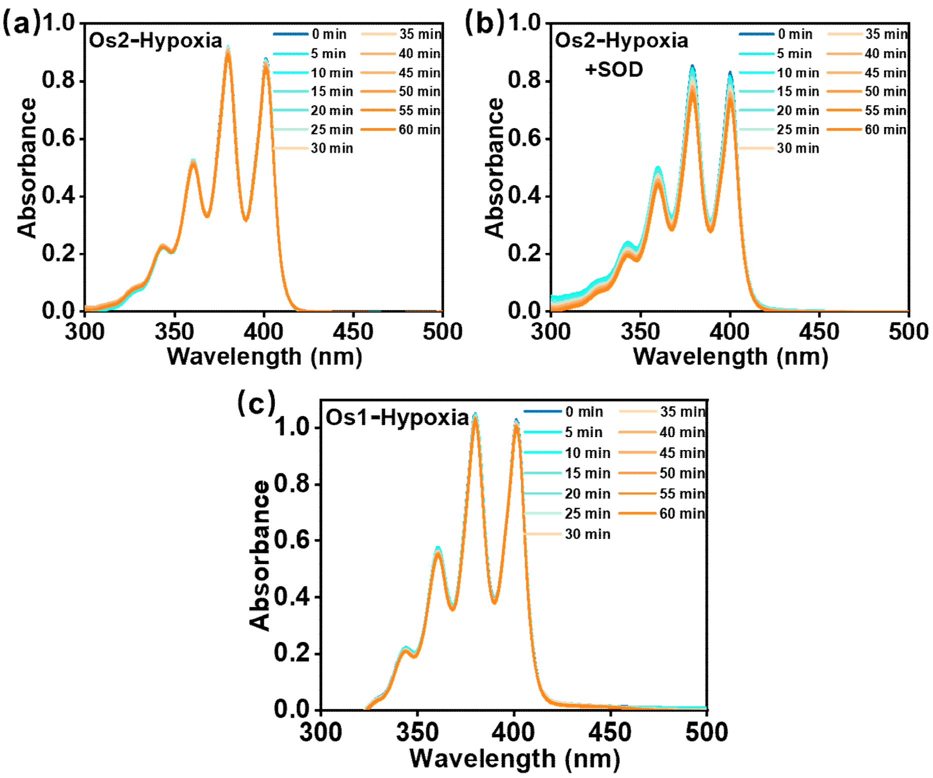

Supplementary Figure 15. The UV-Vis absorption spectra of ABDA (100 μM) for monitoring of $^1O_2$ generation under hypoxia at 298 K. (a) ABDA+**Os2**+hypoxia; (b) ABDA+**Os2**+SOD+hypoxia; (c) ABDA+**Os1**+hypoxia. Light: 465 nm, 13 mW/cm^2; **Os1** or **Os2**: 20 μM.

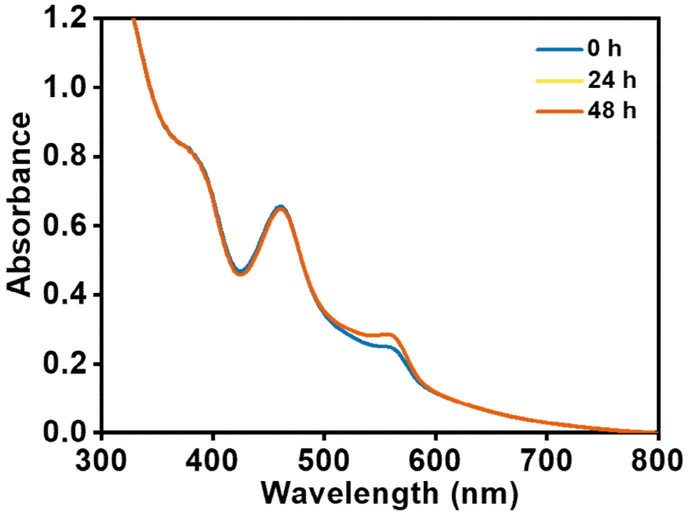

Supplementary Figure 16. The UV-Vis absorption spectra of **Os2** (100 μM) in DMEM medium in the dark at 298 K.

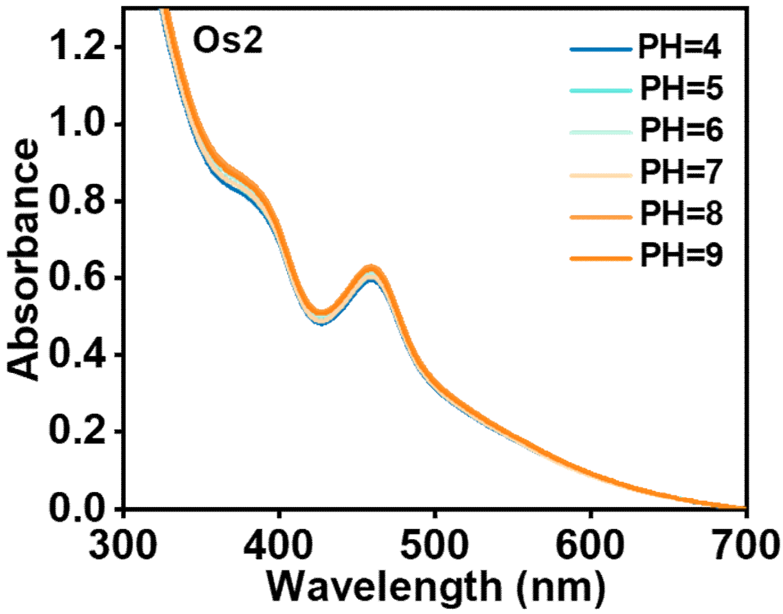

Supplementary Figure 17. The UV-Vis absorption spectra of **Os2** (100 μM) in the PBS solution with different pH values at 298 K.

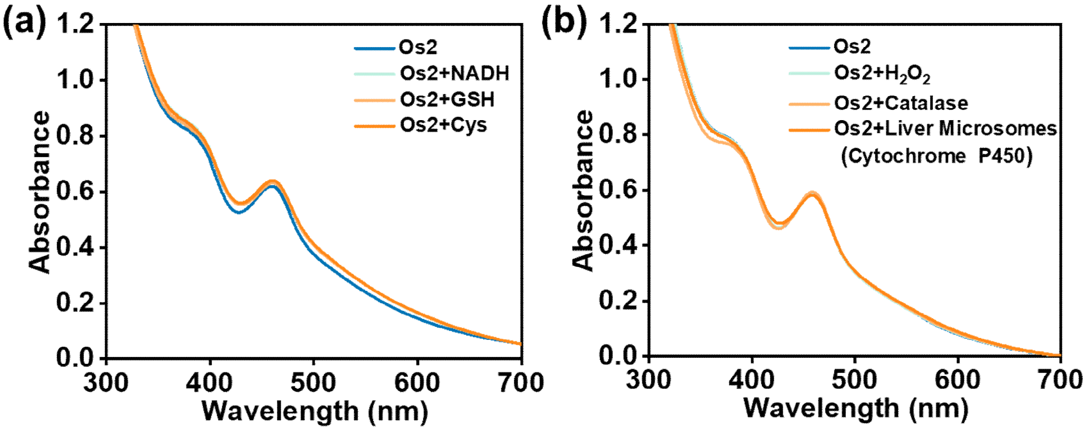

Supplementary Figure 18. (a) The UV-Vis absorption spectra of **Os2** (100 μM) in the presence of cellular reductive biomolecules (50 μM NADH, GSH or Cys) in the dark at 298 K. (b) The UV-Vis absorption spectra of **Os2** (100 μM) in the presence of cells oxidants (50 μM H_2O_2, 500 μg/mL Catalase, Liver Microsomes with 50 nM Cytochrome P450) in the dark at 298 K.

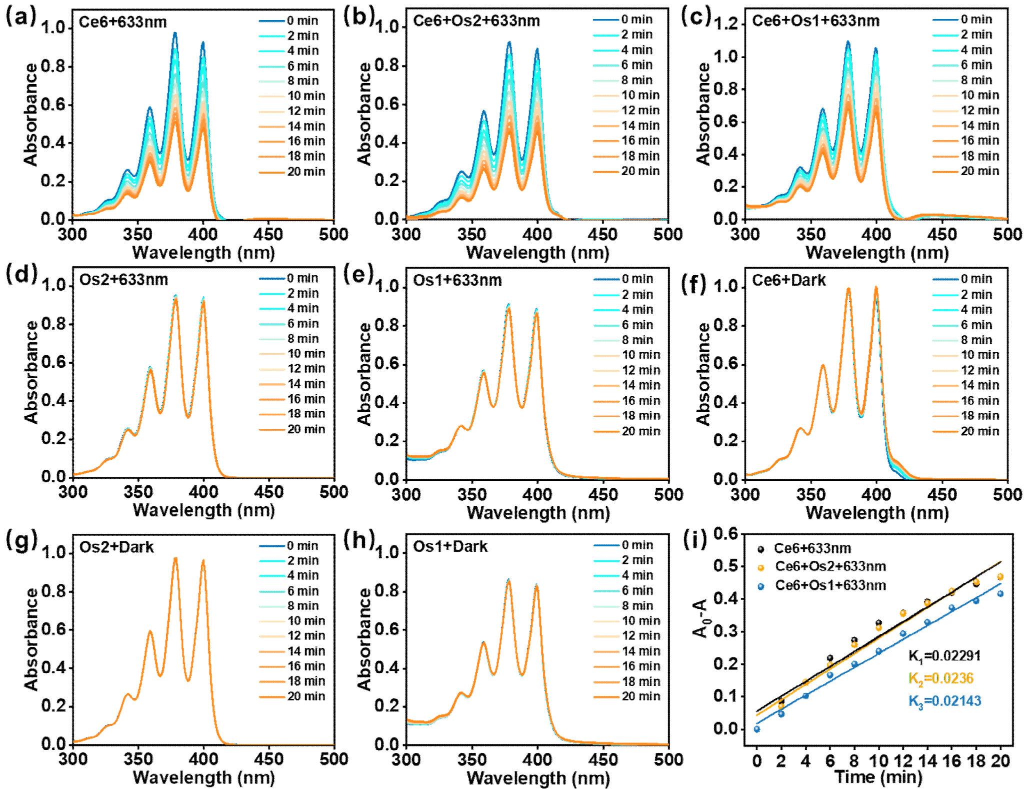

Supplementary Figure 19. (a-e) The UV-Vis absorption spectra of ABDA in the presence of (a) Ce6, (b) Ce6+**Os2**, (c) Ce6 +**Os1**, (d) **Os2** or (e) **Os1** for monitoring of $^1O_2$ generation under 633 nm light irradiation. (f-h) The UV-Vis absorption spectra of ABDA in the presence of (f) Ce6, (g) **Os2** or (h) **Os1** for monitoring of $^1O_2$ generation in the dark. (i) Rate constant for $^1O_2$ generation according to the absorbance of ABDA at 378 nm in (a-c). **Ce6**: 5 μM; **Os2** or **Os1**:10 μM; Light: 633 nm, 6.5 mW/cm^2.

Ce6 can produce $^1O_2$ under 633 nm red light irradiation. However, **Os2** cannot generate $^1O_2$ under 633 nm red light irradiation. When we added both **Os2** and Ce6 in the ABDA solution and then the mixture was irradiated under 633 nm light. The results show that the absorption of ABDA decreases in Ce6 + 633 nm + **Os2** group, which is basically the same as that in Ce6 + 633 nm group, indicating that the addition of **Os2** do not react with (or quench) $^1O_2$. A similar result occurs with **Os1**.

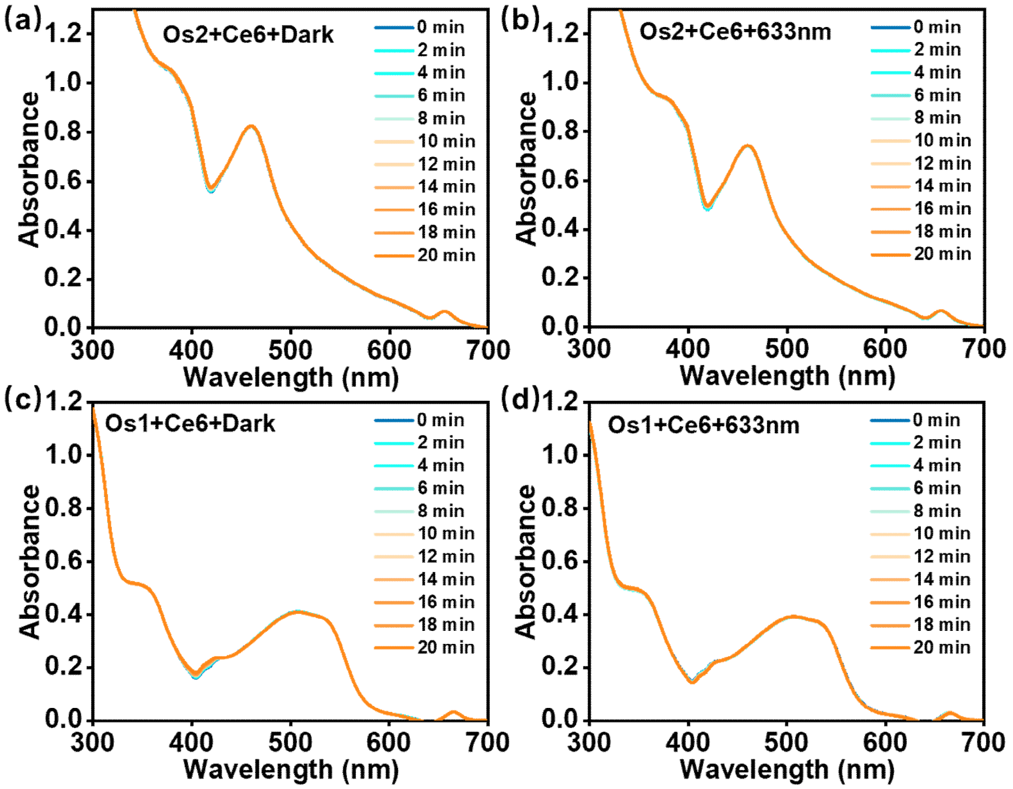

Supplementary Figure 20. (a, c) The UV-Vis absorption spectra of **Os2** or **Os1** (100 μM) in PBS solution (pH 7.4) containing Ce6 (5 μM) in the dark at 298 K. (b, d) The UV-Vis absorption spectra of **Os2** or **Os1** (100 μM) in PBS solution (pH 7.4) containing Ce6 (5 μM) under light irradiation at 298 K. Light: 633 nm, 6.5 mW/cm^2.

Ce6 was added to the **Os2** solution and irradiated with 633 nm light, and then the UV-Vis absorption curves at different irradiation times were measured. The results show that there are no obvious changes in the absorption spectra of **Os2**, meaning that the $^1O_2$ produced by Ce6 does not react with **Os2**. A similar result occurs with **Os1**.

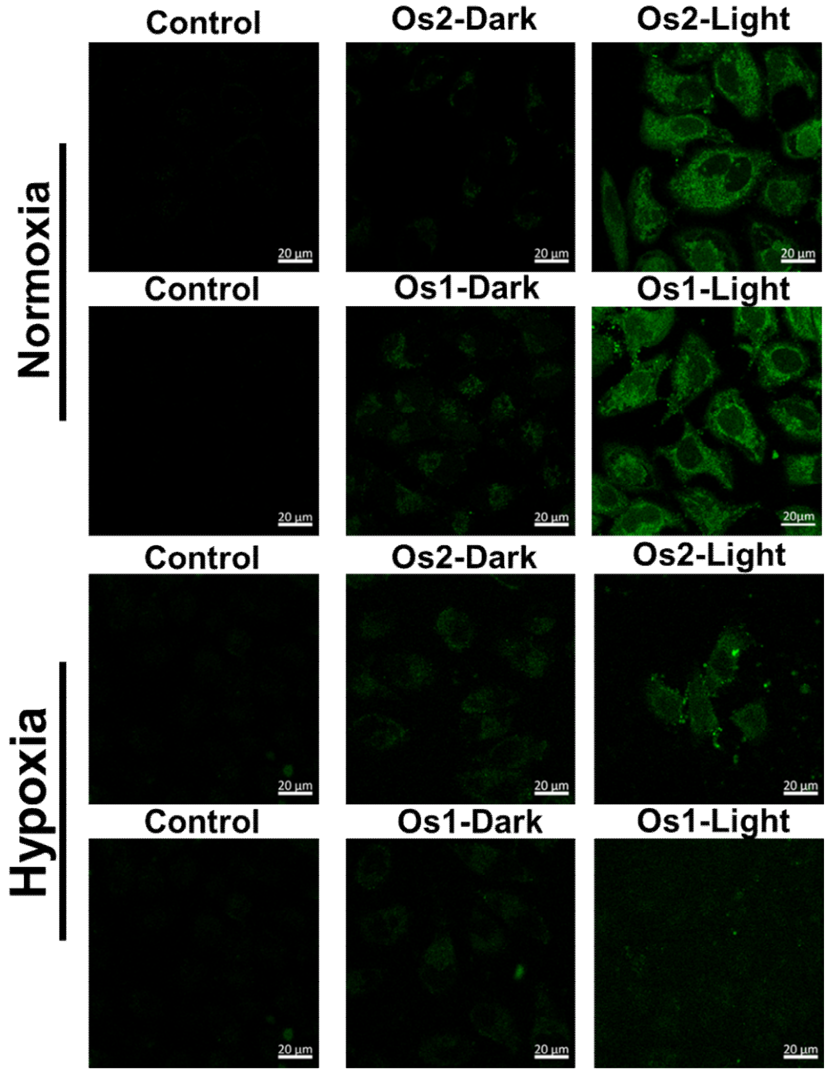

Supplementary Figure 21. Confocal microscopy images of HeLa cells treated with **Os2** or **Os1** (20 μM) and co-stained with SOSG in normoxia or hypoxia at 310 K. The experiment was repeated three times independently with similar results. λ_{ex} = 488 nm, λ_{em} = 525 ± 30 nm. Light irradiation: 465 nm, 13 mW/cm^2, 1 h; Scale bar: 20 μm.

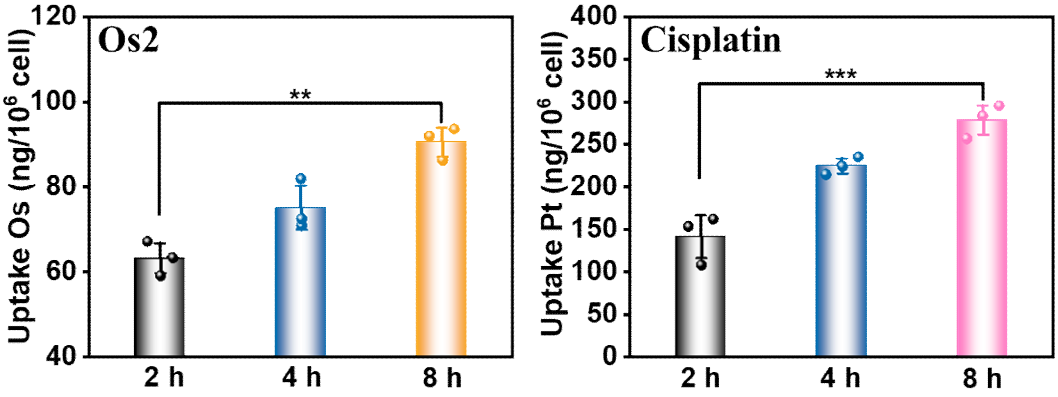

Supplementary Figure 22. Cellular uptake of **Os2** (10 μM) or cisplatin (10 μM) in HeLa cells measured by ICP-MS. All the experiments were performed as duplicates of triplicates (n = 3 biologically independent samples). Error bars represent S.D. from the mean. Statistical significance was calculated with two-tailed Student's t test, $^{**}p$ = 0.0011, $^{***}p = 0.00024$ ($^{*}p < 0.05$, $^{**}p \leq 0.01$ or $^{***}p \leq 0.001$).

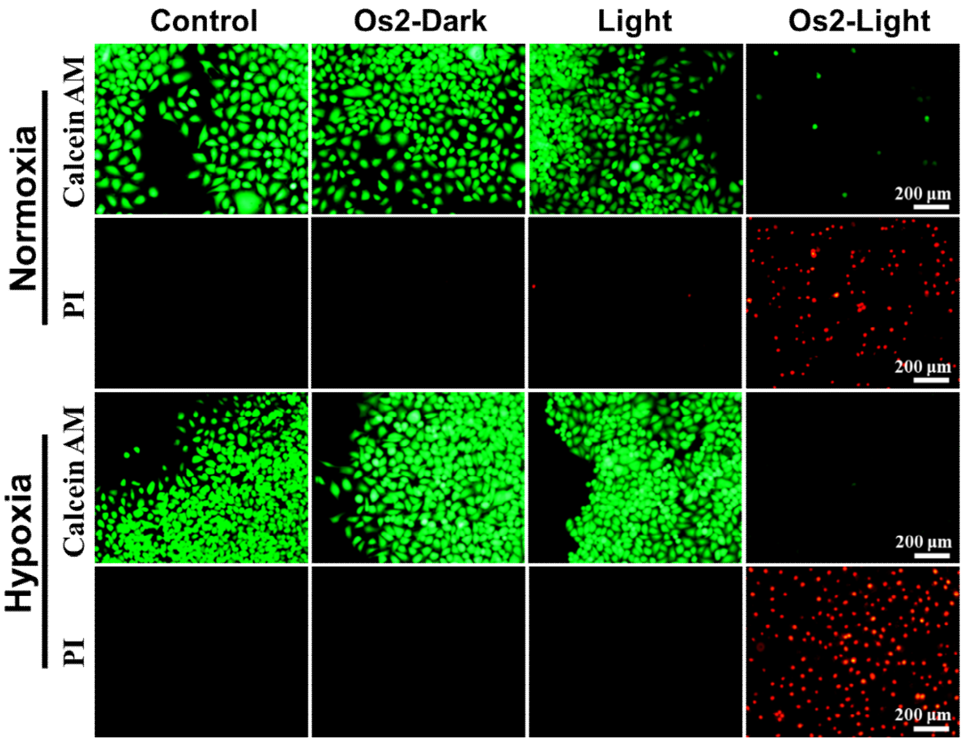

Supplementary Figure 23. Fluorescence microscopy images of the HeLa cells treated with **Os2** (20 μM) and co-stained with calcein AM (green, live cells) (4 μM, 0.5 h) and propidium iodide (PI, red, dead cells) (6 μM, 0.5 h) after different treatments at 310 K. The experiment was repeated three times independently with similar results. Light irradiation: 465 nm, 13 mW/cm^2, 1 h; Calcein AM: $\lambda_{ex} = 460$ nm, $\lambda_{em} = 540 \pm 30$ nm;

PI: λ_{ex} = 540 nm, λ_{em} = 610 ± 30 nm. Scale bar: 200 µm.

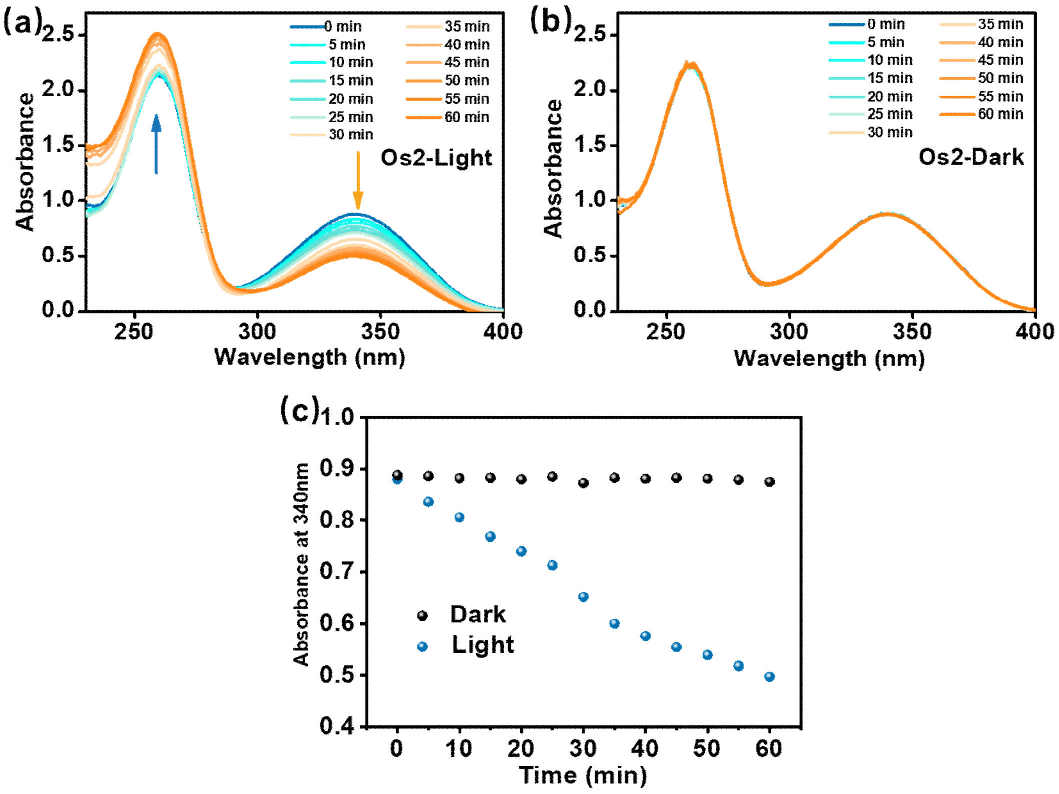

Supplementary Figure 24. The UV-Vis absorption spectra for the photocatalytic oxidation of NADH (175 µM) by **Os2** (20 µM) under light irradiation (a) or in the dark (b) at 298 K. (c) Time dependence of Abs at 340 nm of NADH in (a) and (b). Light irradiation: 465 nm, 13 mW/cm^2.

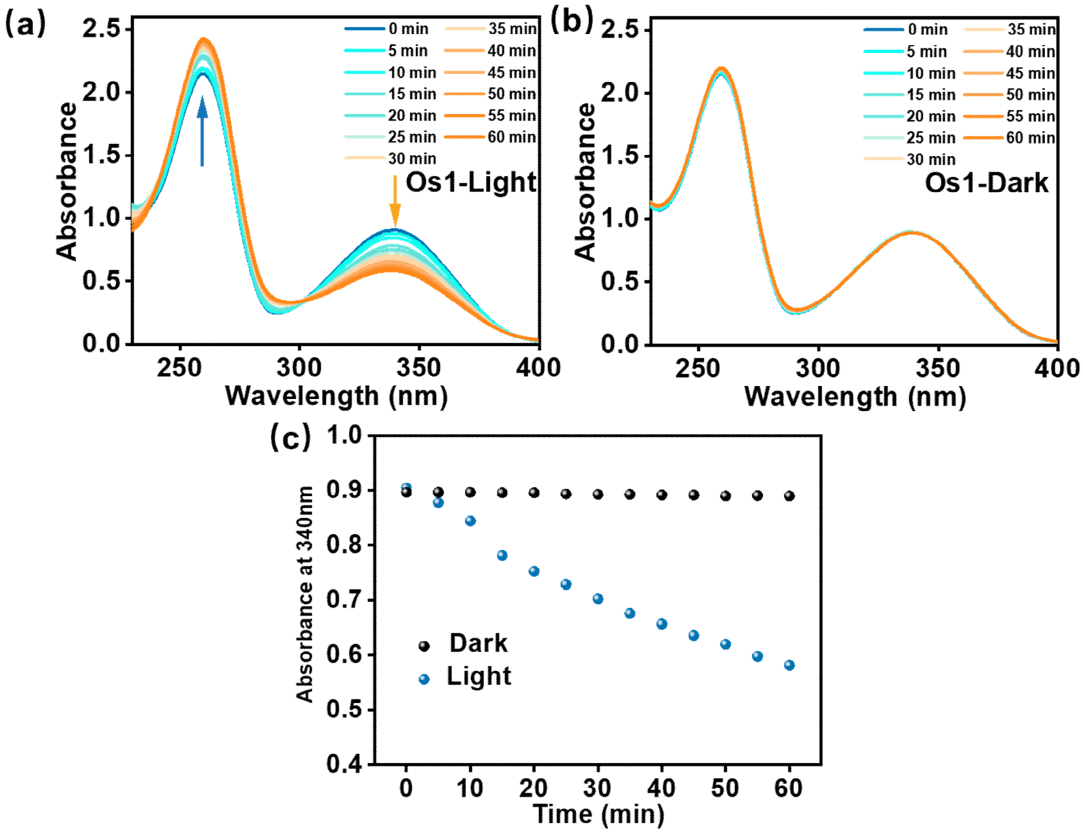

Supplementary Figure 25. The UV-Vis absorption spectra for the photocatalytic oxidation of NADH (175 µM) by **Os1** (20 µM) under light irradiation (a) or in the dark (b) at 298 K. (c) Time dependence of Abs at 340 nm of NADH in (a) and (b). Light irradiation: 465 nm, 13 mW/cm^2.

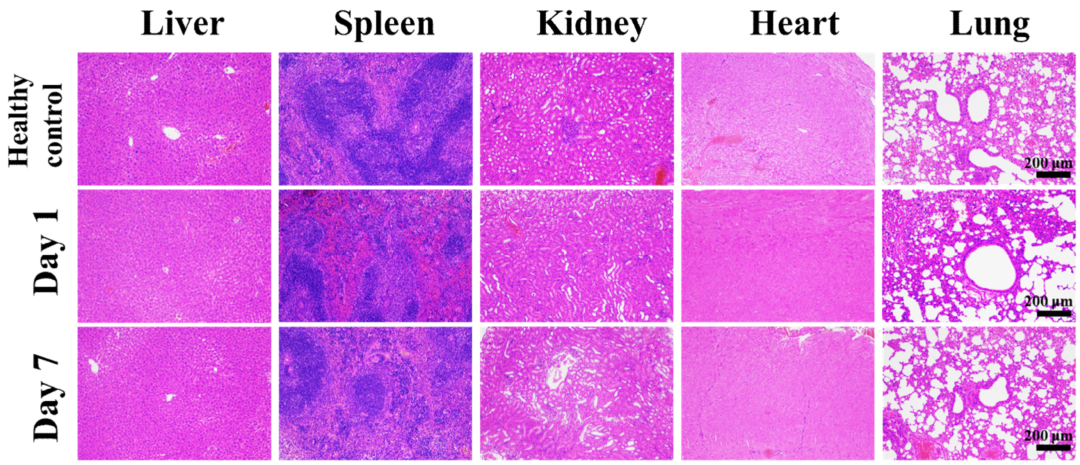

Supplementary Figure 26. H&E staining images of the major organs (heart, liver, spleen, lung, and kidney) of healthy Balb/c mice after i.v. injection of **Os2** (2.69 mg kg^{-1}) at different time points (day 1 and 7). The experiment was repeated three times independently with similar results. Scale bar: 200 µm.

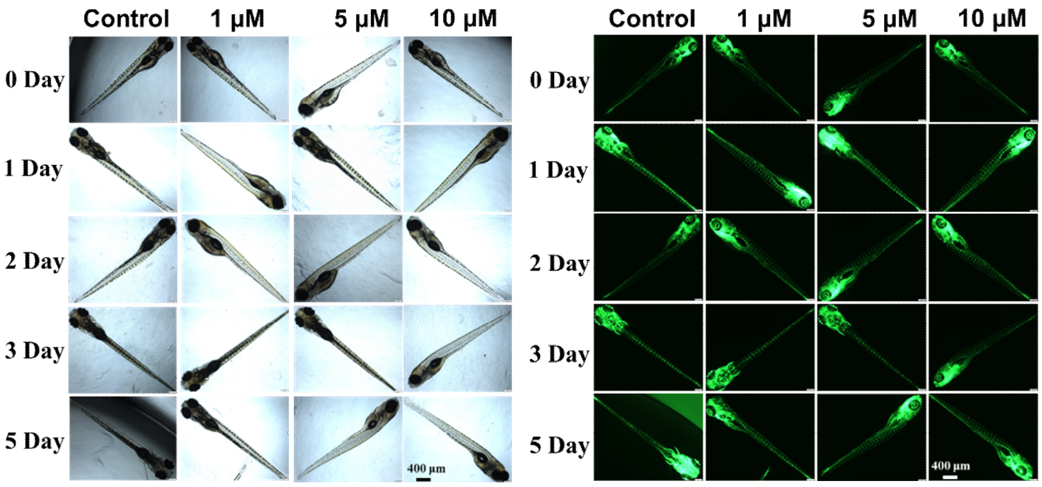

Supplementary Figure 27. Left: zebrafish larvae after treatment with complex **Os2** (1, 5 and 10 µM) for 5 days (monitored by fluorescence microscopy every day). Right: The green fluorescence images were GFP protein expression. λ_{ex} = 460 nm; λ_{em} = 510-550 nm. Scale bar: 400 µm. Experiment temperature: 298 K.

Supplementary Tables

Supplementary Table 1. Selected bond lengths and bond angles for **Os1**.

Bond Distances (Å)					
Os1−O1	2.440(3)	Os1−C1	1.966(5)	Os1−C4	2.095(5)
Os1−C7	1.992(5)	C1−O1	1.254(6)	C1−C2	1.412(7)
C2−C3	1.389(8)	C3−C4	1.407(7)	C4−C5	1.393(7)
C5−C6	1.408(7)	C6−C7	1.361(6)	Os1−Cl1	2.4147(11)

Bond Angles (º)			
Os1−C1−O1	96.0(3)	Os1−C1−C2	127.0(4)
C1−C2−C3	108.0(4)	C2−C3−C4	113.7(5)
Os1−C4−C3	119.0(4)	C4−Os1−C1	72.1(2)
Os1−C4−C5	118.5(4)	C4−C5−C6	112.3(4)
C5−C6−C7	111.7(4)	Os1−C7−C6	124.1(4)
C7−Os1−C4	73.29(19)	C1−Os1−O1	30.75(16)

Supplementary Table 2. Crystal data and structure refinement for **Os1**.

	**Os1**
Empirical formula	$C_{63}H_{51}Cl_2O_3OsP_3$
Formula weight	1210.05
Temperature/K	100.01(10)
Crystal system	triclinic
Space group	P-1
a/Å	12.4529(3)
b/Å	20.3796(6)
c/Å	22.6323(7)
α/°	86.738(3)
β/°	81.319(2)
γ/°	89.640(2)
V/Å^3	5668.7(3)

Z	4
ρ_{calcd}g/cm^3	1.418
μ/mm^{-1}	2.473
F(000)	2432.0
Crystal size/mm^3	0.352 × 0.262 × 0.123
Radiation	MoKα (λ = 0.71073)
2θ range for data collection/°	3.864 to 61.636
Index ranges	-15 ≤ h ≤ 17, -29 ≤ k ≤ 28, -31 ≤ l ≤ 30
Reflections collected	64134
Independent reflections	30084 [Rint = 0.0390, Rsigma = 0.0676]
Data/restraints/parameters	30084/0/1221
Goodness-of-fit on F^2	1.040
Final R indexes [I>=2σ (I)]	R1 = 0.0469, wR2 = 0.1015
Final R indexes [all data]	R1 = 0.0726, wR2 = 0.1167
Largest diff. peak/hole / e Å^{-3}	2.13/-1.98

Supplementary Table 3. Singlet oxygen quantum yields (Φ) of **Os1** and **Os2** by comparison with the $[Ru(bpy)_3]Cl_2$ under the same condition.

Complexes	Φ ($^1O_2$)
$[Ru(bpy)_3]^{2+}$	0.22
**Os2**	0.040±0.0045
**Os1**	0.039±0.0047

Supplementary References

1 Z. Deng, P. Wu, Y. Cai, Y. Sui, Z. Chen, H. Zhang, B. Wang, H. Xia, *iScience*, **2020**, *23*, 101379.

2 C. Zhu, S. Li, M. Luo, X. Zhou, Y. Niu, M. Lin, J. Zhu, Z. Cao, X. Lu, T. Wen, Z. Xie, P. V. Schleyer, H. Xia, *Nat. Chem.* **2013**, *5*, 698-703.

3 S. W. Jin, D. Beis1, T. Mitchell1, J. N. Chen, D. Y. R. Stainier1, *Development*, **2005**, *132*, 5199-5209.
