# Supplementary material for: An osmium-peroxo complex for photoactive therapy of hypoxic tumors
Source: Nat Commun. 2022 Apr 26;13:2245. doi: 10.1038/s41467-022-29969-z (PMC9042834; doi:10.1038/s41467-022-29969-z)
Supplement: Supplementary file 3 — Reporting Summary [file 41467_2022_29969_MOESM3_ESM.pdf]

nature research

Corresponding author(s): Pingyu Zhang, Haiping Xia

Last updated by author(s): Mar 10, 2022

Reporting Summary

Nature Research wishes to improve the reproducibility of the work that we publish. This form provides structure for consistency and transparency in reporting. For further information on Nature Research policies, see our Editorial Policies and the Editorial Policy Checklist.

Statistics

For all statistical analyses, confirm that the following items are present in the figure legend, table legend, main text, or Methods section.

n/a	Confirmed	
☐	☒	The exact sample size (*n*) for each experimental group/condition, given as a discrete number and unit of measurement
☐	☒	A statement on whether measurements were taken from distinct samples or whether the same sample was measured repeatedly
☐	☒	The statistical test(s) used AND whether they are one- or two-sided<br>*Only common tests should be described solely by name; describe more complex techniques in the Methods section.*
☒	☐	A description of all covariates tested
☐	☒	A description of any assumptions or corrections, such as tests of normality and adjustment for multiple comparisons
☐	☒	A full description of the statistical parameters including central tendency (e.g. means) or other basic estimates (e.g. regression coefficient) AND variation (e.g. standard deviation) or associated estimates of uncertainty (e.g. confidence intervals)
☐	☒	For null hypothesis testing, the test statistic (e.g. *F*, *t*, *r*) with confidence intervals, effect sizes, degrees of freedom and *P* value noted<br>*Give P values as exact values whenever suitable.*
☒	☐	For Bayesian analysis, information on the choice of priors and Markov chain Monte Carlo settings
☒	☐	For hierarchical and complex designs, identification of the appropriate level for tests and full reporting of outcomes
☒	☐	Estimates of effect sizes (e.g. Cohen's *d*, Pearson's *r*), indicating how they were calculated

Our web collection on statistics for biologists contains articles on many of the points above.

Software and code

Policy information about availability of computer code

Data collection	NMR spectra were recorded on a BrukerAV III-400/600 MHz spectrometer. HRMS measurements were conducted using Thermo Scientific Q Exactive instrument. Single-crystal X-ray diffraction data were collected using a Bruker APEX-II CCD diffractometer with CuKα radiation (λ = 1.54178 Å). Elemental analyses were performed on a Vario EL III elemental analyzer. UV-visible absorption spectra were recorded on a Hitachi UV-2500 spectrophotometer. The emission spectra were recorded on an Edinburgh FS5 Spectrofluorometer. HPLC spectra was recorded on Agilent 1260 high performance liquid chromatograph. ESR spectra were recorded using a Bruker Model A300 ESR spectrometer equipped with a Bruker ER 4122 SHQ resonator. Confocal images were recorded on a Zeiss LSM 880 confocal microscopy. The cell viability assays were recorded using a Promega microplate reader.
Data analysis	All ploted and calculated statistical analyses were performed on Excel 2019 and Origin 8.5.

For manuscripts utilizing custom algorithms or software that are central to the research but not yet described in published literature, software must be made available to editors and reviewers. We strongly encourage code deposition in a community repository (e.g. GitHub). See the Nature Research guidelines for submitting code & software for further information.

Data

Policy information about availability of data

All manuscripts must include a data availability statement. This statement should provide the following information, where applicable:

- Accession codes, unique identifiers, or web links for publicly available datasets
- A list of figures that have associated raw data
- A description of any restrictions on data availability

Data availability
The authors declare that all data needed to evaluate the conclusion of this work are presented in the paper, the supplementary information or source data file. The

source data have been deposited in the Figshare database under accession code DOI:10.6084/m9.figshare.19333802 [https://figshare.com/articles/figure/Daet_of_An_osmium-peroxo_complex_for_photoactive_therapy_of_hypoxic_tumors_/19333802]. Source data are also provided with this paper. The crystal structure of Os1 was deposited in the Cambridge Crystallographic Data Centre (CCDC 1913382).

Field-specific reporting

Please select the one below that is the best fit for your research. If you are not sure, read the appropriate sections before making your selection.

☒ Life sciences ☐ Behavioural & social sciences ☐ Ecological, evolutionary & environmental sciences

For a reference copy of the document with all sections, see nature.com/documents/nr-reporting-summary-flat.pdf

Life sciences study design

All studies must disclose on these points even when the disclosure is negative.

Sample size	Sample size for in vitro cytotoxicity assays were duplicates of quadruplicate with the same number of the cells. The in vivo group sizes (5 animals per treatment group, the mice is the same age and sex with almost same weight and health) represents the minimum number animals needed to reach statistical significance ($p < 0.05$) between experimental groups. Statistical analyses (two tailed t-test with unequal variances) were carried out assuming the correct sample size. No statistical methods were used to predetermine sample sizes. All of our experiments followed well established reported protocols.
Data exclusions	No data were excluded.
Replication	Experiments were repeated and experimental findings were reproducible. Details of experimental replicates are given in the figure legends or methods.
Randomization	All experimental samples of mice were allocated randomly to each group. No randomization was used for in cellular experiments because the number of cells are the same.
Blinding	Blinding were applied to all mice experiments. Blinding is not possible as all our in vitro experiments are based on cell culture.

Reporting for specific materials, systems and methods

We require information from authors about some types of materials, experimental systems and methods used in many studies. Here, indicate whether each material, system or method listed is relevant to your study. If you are not sure if a list item applies to your research, read the appropriate section before selecting a response.

Materials & experimental systems

n/a	Involved in the study	
☐	☒	Antibodies
☐	☒	Eukaryotic cell lines
☒	☐	Palaeontology and archaeology
☐	☒	Animals and other organisms
☒	☐	Human research participants
☒	☐	Clinical data
☒	☐	Dual use research of concern

Methods

n/a	Involved in the study	
☒	☐	ChIP-seq
☒	☐	Flow cytometry
☒	☐	MRI-based neuroimaging

Antibodies

Antibodies used	β-Actin Rabbit Monoclonal Antibody (catalog number:AF5003) and GPX4 Rabbit Polyclonal Antibody (catalog number AF7020) were bought from Beyotime Company, China
Validation	The product is limited to the scientific research of professionals and cannot be used for clinical diagnosis or treatment. The validation can be seen on the website:https://www.beyotime.com/Manual/AF7020%20GPX4%20Rabbit%20Polyclonal%20Antibody.pdf

Eukaryotic cell lines

Policy information about cell lines

Cell line source(s)	HeLa Cervical cancer cell line was obtained from American type culture collection (ATCC).
Authentication	The cell line we used was morphologically confirmed according to the information provided by ATCC.

Mycoplasma contamination	All cell lines were tested for mycoplasma contamination. No mycoplasma contamination was found.
Commonly misidentified lines (See ICLAC register)	No commonly misidentified cell lines are used in this study.

Animals and other organisms

Policy information about studies involving animals; ARRIVE guidelines recommended for reporting animal research

Laboratory animals	Female BALB/c mice (6-8 weeks) were used as animal model in this work and were purchased from Liaoning Changsheng Biotechnology Co. Ltd. Mice were housed in individually ventilated cage (IVC) systems (ambient temperature: 23 ± 3 °C; relative humidity: 40-70%) and exposed to a 12-h light–dark cycle with free access to food and water.<br>The zebrafish (Tg(flk1:EGFP)s843, <5 dpf) was obtained from Center of Experiment Animals at Sun Yat-Sen University. All zebrafish has been transfected by GFP. Embryos and zebrafish were housed in circular petri dish (Case of 150) at 28 ± 2 °C, and monitored at least once a day.
Wild animals	The study did not involve wild animals.
Field-collected samples	The study did not involve samples collected from the field.
Ethics oversight	The experimental protocols were approved by the Experimental Animal Ethics Committee of the Health Science Center of Shenzhen University (Shenzhen, China). All animal experimental procedures were performed in accordance with the Regulations for the Administration of Affairs Concerning Experimental Animals approved by the State Council of the People's Republic of China.

Note that full information on the approval of the study protocol must also be provided in the manuscript.
